# G719X/L861Q/S768I突变非小细胞肺癌诊断及靶向治疗进展

**DOI:** 10.3779/j.issn.1009-3419.2024.101.20

**Published:** 2024-08-20

**Authors:** Yufang WANG, Jing ZHENG, Yanping ZHU, Jianya ZHOU

**Affiliations:** 310000 杭州，浙江大学医学院附属第一医院呼吸与危重症医学科; Department of Respiratory Medicine, Thoracic Disease Center, the First Affiliated Hospital, College of Medicine, Zhejiang University, Hangzhou 310000, China

**Keywords:** 肺肿瘤, 表皮生长因子受体, 主要罕见突变, 靶向治疗, Lung neoplasms, Epidermal growth factor receptor, Major rare mutations, Targeted therapy

## Abstract

肺癌在全球癌症死亡原因中占比最高，对人类健康造成了极大威胁。30%-40%的非小细胞肺癌（non-small cell lung cancer, NSCLC）的发生是由于表皮生长因子受体（epidermal growth factor receptor, EGFR）发生点突变 、外显子插入、外显子缺失导致。除常见的19号外显子缺失突变和21号外显子L858R突变外，18号外显子G719X突变、21号外显子L861Q突变、20号外显子S768I突变是最主要的罕见突变。目前，针对主要罕见突变的诊断方法主要是下一代测序技术（next-generation sequencing, NGS ）、数字聚合酶链式反应（digital polymerase chain reaction, dPCR）、微滴式数字PCR（droplet digital PCR, ddPCR）等。关于G719X/L861Q/S768I突变NSCLC的靶向治疗，第一代EGFR酪氨酸激酶抑制剂（tyrosine kinase inhibitors, TKIs）疗效较差，第二代和第三代EGFR-TKIs疗效相当，新型第三代EGFR-TKIs和联合治疗展现出不错的治疗前景。本文对G719X/L861Q/S768I突变NSCLC诊断及靶向治疗进展进行了归纳，以期为后续临床用药及研究提供参考。

## Abstract (en)

Lung cancer accounts for the highest proportion of cancer deaths in the world and poses a great threat to human health. About 30% to 40% of non-small cell lung cancer (NSCLC) is caused by point mutations, exon insertion and exon deletion of the epidermal growth factor receptor (EGFR). In addition to the common exon 19 deletion mutation and exon 21 L858R mutation, exon 18 G719X mutation, exon 21 L861Q mutation and exon 20 S768I mutation are the most important rare mutations. At present, the diagnostic methods for major rare mutations are mainly next-generation sequencing (NGS), digital polymerase chain reaction (dPCR), droplet digital PCR (ddPCR), etc. Regarding the targeted therapy of G719X/L861Q/S768I mutant NSCLC, the first generation EGFR-tyrosine kinase inhibitors (TKIs) have poor efficacy, while the second and third generation EGFR-TKIs have similar efficacy. The novel third generation EGFR-TKIs and combination therapy show a good therapeutic prospect. This article summarized the progress in the diagnosis and targeted therapy of G719X/L861Q/S768I mutant NSCLC, so as to provide reference for subsequent clinical drug use and research.

肺癌是包括中国在内的世界范围内癌症死亡的主要因素之一。Freddie Bray团队^[1]^发布的数据表明，全球每年约有248万新发肺癌病例，导致超过181.7万人死亡。2022年，我国共报告肺癌新发病例106.06万，死亡病例73.33万^[2]^。非小细胞肺癌（non-small cell lung cancer, NSCLC）是肺癌的主要类型，占肺癌患者的80%-85%，其中腺癌是最常见的亚型。作为驱动癌基因，表皮生长因子受体（epidermal growth factor receptor, EGFR）突变在亚洲NSCLC患者中的发生率高于50%^[3,4]^。EGFR酪氨酸激酶抑制剂（tyrosine kinase inhibitors, TKIs）靶向治疗对此类突变疗效较好。随着基因检测技术的进展，越来越多的EGFR罕见突变被发现，而这些突变对EGFR-TKIs的反应具有异质性，相关诊断及治疗需要进一步的研究。本文对近年来NSCLC中主要罕见突变（G719X/L861Q/S768I）的诊断及靶向治疗进展进行了相关综述，以期为临床用药及后续研究提供参考。

## 1 EGFR与NSCLC

EGFR是由Erb B基因编码的跨膜糖蛋白，含有上千个氨基酸，是一种广泛分布于哺乳动物细胞的跨膜受体，其信号通路与肿瘤细胞的增殖和转移、血管生成和凋亡密切相关。其结构由3个部分组成：胞外结构域（N端）、穿膜结构域（疏水的α螺旋结构）和胞内结构域（C端）。胞外域包含622个氨基酸，由4个亚结构域组成：I结构域（氨基酸1-151位）、II结构域（氨基酸152-312位）、III结构域（氨基酸313-481位）和IV结构域（氨基酸482-621位）。胞内结构域含有542个氨基酸，由3个亚结构域组成：近膜端结构域（约含有50个氨基酸）、酪氨酸激酶结构域（含有250个氨基酸）和C末端尾部（含有229个氨基酸），C末端尾部包含5个自磷酸化基序^[5]^。

EGFR基因位于人第7号染色体的短臂p12-13上，由188,307个碱基^[6]^和28个外显子组成，属于ERBB家族^[5]^。在亚洲人群中，50%以上的肺腺癌可以检测到EGFR突变^[4]^。EGFR的酪氨酸激酶功能域由18-24个外显子编码。迄今为止，已有超过30种EGFR突变被检测并报道^[6]^。其中大多数EGFR突变为19号外显子缺失突变和21号外显子L858R点突变，二者统称为常见EGFR突变，占总EGFR突变的80%-90%^[7]^。由于下一代测序技术（next-generation sequencing, NGS）等基因检测技术的广泛应用，使得罕见EGFR突变的检出率越来越高。根据目前统计，有10%-15%的NSCLC存在罕见EGFR突变，其中G719X、L861Q、S768I为主要的罕见突变，分别占所有EGFR突变的~3%、~1%、~1%^[6]^。G719X是发生率最高的18号外显子突变，也是仅次于20号外显子插入的罕见突变；L861Q突变是除L858R突变外最常见的21号外显子突变；而S768I突变是最具特征性的20号外显子突变之一^[8]^。

## 2 G719X/L861Q/S768I突变的诊断进展

在NSCLC中，依据结构的不同将EGFR突变分为4种类型，分别为远离腺嘌呤核苷三磷酸（adenosine triphosphate, ATP）结合口袋的经典样突变、T790M样疏水核心突变、20号外显子的αC-螺旋末端的C端插入环突变及P环αC螺旋压缩（P-loop αC-helix compression, PACC）突变。其中，L861Q突变的位点远离ATP结合口袋，属于经典样突变。而G719X和S768I则均属于PACC突变^[9]^。EGFR基因检测可作为分子靶向治疗的指导，有助于靶向治疗药物及抗耐药抑制剂的研发。本文对NSCLC患者中EGFR基因的检测进展进行了综述，并在表1^[10⇓⇓⇓⇓⇓⇓⇓⇓⇓⇓⇓⇓-23]^及表2^[16,23⇓⇓-26]^中列举了近年来EGFR检测的常用方法。

**表1:** G719X/L861Q/S768I突变NSCLC的组织活检

Author	EGFR status	n	Method
Shen et al.^[10]^	S768I/L861Q	2	Sanger sequencing
Carvalho et al.^[11]^	G719X/S768I/L861Q	6	dPCR
Guo et al.^[12]^	G719X	33	NGS
	L861Q	4	NGS
	S768I	17	NGS
Colling et al.^[13]^	L861Q	1	NGS
	S768I	1	NGS
He et al.^[14]^	G719X	62	ARMS-PCR
	L861Q	47	ARMS-PCR
	S768I	26	ARMS-PCR
Ma et al.^[15]^	G719X	5	NGS
	L861Q	3	NGS
	G719X+L861Q	2	NGS
	G719X+S768I	22	NGS
He et al.^[16]^	G719X	2	NGS
	L861Q	3	NGS
	S768I	2	NGS
Lechner et al.^[17]^	L861Q	9	ddPCR
Ke et al.^[18]^	G719X	5	ARMS-PCR
	L861Q	14	ARMS-PCR
	G719X+S768I	2	ARMS-PCR
Chai et al.^[19]^	L861Q	2	ARMS-PCR
Mack et al.^[20]^	G719X	18	NGS
Zhao et al.^[21]^	L861Q	5	NGS
Jakobsen et al.^[22]^	S768I	1	NGS
Visser et al.^[23]^	S768I	1	NGS

EGFR: epidermal growth factor receptor; NSCLC: non-small cell lung cancer; dPCR: digital polymerase chain reaction; ddPCR: droplet digital PCR; ARMS-PCR: amplification refractory mutation system PCR; NGS: next-generation sequencing.

**表2:** G719X/L861Q/S768I突变NSCLC的液体活检

Author	EGFR status	n	Method
He et al.^[16]^	G719X	1	NGS
	L861Q	3	NGS
	S768I	2	NGS
Visser et al.^[23]^	L861Q	1	ddPCR
Xu et al.^[24]^	G719X	1	ARMS-PCR
	G719X	1	ddPCR
	G719X	1	NGS
Lei et al.^[25]^	L861Q	5	NGS
	S768I	7	NGS
	G719X+S768I	3	NGS
Buder et al.^[26]^	L861Q	6	ddPCR
	G719X+S768I	1	ddPCR

### 2.1 组织活检

组织活检是目前肺癌诊断的金标准。临床上获取组织标本的方法主要有支气管内超声引导细针穿刺活检、外科手术、经皮穿刺肺活检、胸腔镜穿刺活检、胸水细胞检测等^[27]^。

目前存在多种检测EGFR突变的方法，临床上常用的方法主要有基于聚合酶链式反应（polymerase chain reaction, PCR）的技术平台、基于测序技术的Sanger测序和NGS。

PCR技术目前已获得长足发展。从传统PCR到基于实时荧光定量PCR技术的扩增阻滞突变系统PCR（amplification refractory mutation system PCR, ARMS-PCR）到数字PCR（digital PCR, dPCR），其灵敏度和特异性不断提高。ARMS-PCR提供的检测结果虽然较为准确可靠，但可能会忽略罕见突变，Shen等^[10]^的研究对100例ARMS-PCR检测为EGFR野生型的样本进行Sanger测序复检，其中24例测序失败，2例被排除，余下74例样本中检出了2例S768I/L861Q突变。Carvalho等^[11]^对dPCR和实时PCR的灵敏度和特异性进行了比较，发现dPCR的定量限为1%，非常适合用于低丰度突变的评估及检测和定量罕见突变。但PCR技术最重要的限制是只能检测个别突变，不能对患者进行全面的基因分型。

基于边合成边测序原理的NGS技术具有通量高、检测突变位点类型多样等优点，越来越多地应用于临床肺癌靶向基因检测^[12]^。Colling等^[13]^对NGS和基于实时定量PCR的Idylla^TM^技术进行比较，结果显示，被Idylla^TM^检测为野生型的2例样本在NGS中分别被检出L861Q、S768I突变。He等^[14]^的研究对3665及802例组织样本分别进行了ARMS-PCR检测、NGS检测，通过比较说明NGS具有更高的通量和更广泛的检测范围，在检测多突变和罕见突变方面具有显著优势。但进行NGS检测的设备和试剂费用昂贵，难以在我国基层医院推广。

Obradovic等^[28]^对已发表的987篇文献进行综合分析，表明在亚洲地区使用最广泛的EGFR检测方法是PCR技术平台，Sanger测序次之。而在全球范围内，NGS的使用频率在逐年攀升。目前还没有真正的“一刀切”基因检测方法，临床医生和检测人员需要在实践中考虑到成本、周转时间、检测结果质量等因素，从多样化的检测方法中合理选择，必要时可采用不同方法相互验证补充^[29]^。

### 2.2 液体活检

作为肺癌诊断的金标准，组织活检存在操作复杂、侵入性强、对患者不良反应较大的劣势。近年来兴起的液体活检以相对无创的方式，能更加方便快捷地监测肺癌的进展、治疗反应和预后情况^[30,31]^。并且有研究^[15]^证实，液体活检可应用于重复活检，对分子变化进行动态监测，这对晚期肺癌患者及耐药患者来说具有重大意义。通过液体活检分析的成分是独特的肿瘤衍生物：循环肿瘤细胞（circulating tumor cells, CTCs），离开原发部位的癌细胞通过血液侵犯其他组织；细胞游离DNA（cell-free DNA, cfDNA），由Mandel和Metais于1948年首先描述，在肿瘤患者的血清中出现水平升高；循环肿瘤DNA （circulating tumor DNA, ctDNA），是cfDNA的一部分；肿瘤来源的RNAs（即mRNA和miRNA）及细胞外囊泡，如外泌体（exosome）^[31]^。

除血液外，可进行液体分析的体液还包括尿液、唾液、粪便、胸腔积液、脑脊液等^[31]^。Liu等^[32]^的研究表明，在NSCLC脑转移的患者中，脑脊液中ctDNA的丰度和检出率均高于配对血浆。

液体活检的分析前阶段有标本收集、稳定、运输、富集、处理及分析物的分离和质量评估。此过程中的失误会对后续的数据质量产生不利影响，甚至导致错误的检测结果和治疗决策^[30]^。因此，对样本的储存条件、样本采集与处理的间隔时间、样本处理方案、质量评估方法等进行标准的流程化规定十分必要。

目前应用于液体活检的基因检测方法主要有dPCR、微滴式数字PCR（droplet digital PCR, ddPCR）和NGS。有研究^[24]^比较了4种用于液体活检的检测方法，分别为Cobas-ARMS、ADx-ARMS、ddPCR和NGS，结果表明除ADx-ARMS外，另外3种技术均可在等位基因频率>1%的情况下检出G719X突变，且ddPCR和NGS可定量给出突变的丰度，展示更丰富的检测信息。ddPCR是在PCR扩增前对样品进行微滴化处理，于2011年由Bio-Rad公司推出。研究^[33]^表明，在相对丰度分数为0.25%的情况下，其仍可靠地检测到样本的突变浓度，这有利于液体活检对微小残留病灶（minimal residual disease, MRD）的识别^[8]^。He等^[16]^的研究对中国肺癌患者的配对血液和组织样本进行了NGS检测，以探索血液和组织样本检测结果的一致性。结果表明，G719X、S768I、L861Q检测的敏感度分别为50%（n=2）、100%（n=3）、100%（n=2）。还有研究^[25]^对28例患者的配对血液和组织样本采用了NGS检测，其中检出1例G719X，1例L861Q，2例G719X+S768I，1例G719X+L861Q，结果表现出了高度一致性。

需要注意的是，液体活检也有一定的局限性。根据肿瘤负荷的不同，其释放的ctDNA不尽相同，丰度从小于0.01%到大于10%不等。而且用于检测的ctDNA不仅在肿瘤患者中升高，在自身免疫性疾病、创伤、剧烈运动的情况下也可有升高^[15,29,31]^。在大多数早期癌症患者中，ctDNA的量很低，与健康受试者相似，进行检测易出现假阴性。故组织活检和液体活检应相互配合，为治疗过程中的肿瘤演变提供完整画像^[34]^。

## 3 靶向治疗进展

### 3.1 G719X/L861Q/S768I突变与TKIs

EGFR-TKIs治疗是经典EGFR突变NSCLC的标准治疗方案，已在多个国家被批准用于EGFR突变肺癌患者的一线治疗。但现有的临床试验大多局限于经典EGFR突变的患者，截至2021年底，仅有5项EGFR-TKIs的随机试验纳入了少量的罕见突变患者：IPASS（吉非替尼）、NEJ002（吉非替尼）、LUX-Lung 3和6（阿法替尼）、KCSG-LU15-09（奥希替尼）^[35]^。这些临床试验的数据表明，主要罕见突变对EGFR-TKIs的敏感性具有异质性，需要对TKIs进行特殊选择。

### 3.2 第一代TKIs

吉非替尼（Gefitinib）、厄洛替尼（Erlotinib）和埃克替尼（Icotinib）为可逆性EGFR-TKIs，均为喹唑啉的衍生物，它们作为ATP竞争性抑制剂，通过与EGFR的酪氨酸激酶口袋结合发挥作用^[36]^。Popat等^[35]^的研究纳入了G719X（n=46）/L861Q（n=34）/S768I（n=6）共86例接受吉非替尼或厄洛替尼治疗的患者，结果显示第一代TKIs对G719X/L861Q/S768I突变NSCLC有一定疗效，患者经一线治疗后的客观缓解率（objective response rate, ORR）为47.3%，中位总生存期（median overall survival, mOS）为28.5个月。但有研究^[37]^表明G719X/L861Q/S768I突变NSCLC对第一代TKIs的反应与经典突变相比较差，结果显示，G719X、L861Q、S768I、经典突变治疗后的中位无进展生存期（median progression-free survival, mPFS）分别为8.2、7.6、3.4和10.9个月。同时多项研究^[38⇓⇓-41]^表明G719X/L861Q/S768I突变NSCLC经第一代TKIs治疗后的ORR均不超过50%。G719X/L861Q/S768I突变NSCLC对第一代TKIs的敏感性可能差于经典突变NSCLC。Lei等^[25]^通过回顾性研究探索21例埃克替尼原发耐药患者的基因突变图谱，其中检出11例S768I（占比52.4%）、5例L861Q（占比23.8%）、3例G719X（占比14.3%），进一步说明G719X/L861Q/S768I突变 NSCLC对第一代TKIs的反应较差。当然，上述研究均为回顾性研究且样本量较小，可能存在误差导致结果不能切实反映生存情况，但总的来说，G719X/L861Q/S768I突变 NSCLC对第一代TKIs的敏感性较差，见表3^[35,37⇓⇓⇓⇓⇓⇓⇓-45]^。

**表3:** 第一代TKIs在G719X/L861Q/S768I突变NSCLC中的疗效

Author	Year	Drug	Study type	n	EGFR status	ORR (%)	mPFS (mon)	mOS (mon)
Popat et al.^[35]^	2022	Gefitinib/Erlotinib	Retrospective study	80	G719X/L861Q/S768I	47.3		28.5
Shi et al.^[37]^	2017	Gefitinib/Erlotinib/ Icotinib	Retrospective study	27	G719X		8.2	
				17	L861Q		7.6	
				9	S768I		3.4	
Chen et al.^[38]^	2017	Gefitinib/Erlotinib	Retrospective study	19	G719X	37.0		
				16	L861Q	31.0		
				2	S768I	0.0		
Zhang et al.^[39]^	2017	Gefitinib/Erlotinib	Retrospective study	22	G719X	23.0	7.6	
				5	L861Q	0.0	5.7	
				11	S768I	27.0	8.0	
Tu et al.^[40]^	2017	Gefitinib/Erlotinib	Retrospective study	16	G719X	50.0	11.6	25.2
Passaro et al.^[41]^	2019	Gefitinib/Erlotinib	Retrospective study	42	G719X	31.0	8.3	
Ullas et al.^[42]^	2023	Gefitinib	Retrospective study	1	L861Q		5.1	
				1	S768I		7.3	
Zhang et al.^[43]^	2020	Gefitinib/Erlotinib/ Icotinib	Retrospective study	5	G719X	80.0	6.0	
				5	L861Q	40.0	6.2	
Kate et al.^[44]^	2019	Gefitinib/Erlotinib	Retrospective study	5	G719X		8.4	13.5
				2	L861Q		1.0	2.0
				2	S768I		2.0	1.8
Pilotto et al. ^[45]^	2018	Gefitinib/Erlotinib	Retrospective study	6	G719X		8.4	17.0
				5	L861Q		5.2	14.5

TKIs: tyrosine kinase inhibitors; ORR: objective response rate; mPFS: median progression-free survival; mOS: median overall survival.

### 3.3 第二代TKIs

阿法替尼（Afatinib）和达克替尼（Dacomitinib）是第二代EGFR-TKIs，作为一种不可逆的阻断剂，它们与受体的酪氨酸激酶结构域共价结合，导致其失活，并阻止其参与细胞生长和存活的下游信号通路^[7,36]^。

阿法替尼用于治疗EGFR非耐药突变的NSCLC患者，目前已在全球80多个国家获得批准。LUX-Lung3和LUX-Lung6两项随机III期试验^[8]^的合并分析显示，应用阿法替尼治疗EGFR G719X/L861Q/S768I突变NSCLC的mPFS和mOS明显长于化疗组，此项研究说明EGFR G719X/L861Q/S768I突变NSCLC对阿法替尼较为敏感。后续的多项研究均证实了这一结论。一项回顾性研究^[46]^表明，G719X/L861Q/S768I突变NSCLC（n=90）接受阿法替尼治疗后的ORR为63.3%，mPFS为17.3个月，mOS为28.5个月。Yang等^[47]^的研究显示，阿法替尼治疗后的G719X（n=181）、L861Q（n=97）和S768I（n=56）突变NSCLC的ORR分别为61.3%、57.7%和71.4%。另有回顾性研究^[42]^显示，G719X（n=4）、L861Q（n=2）、S768I（n=1）突变NSCLC接受阿法替尼治疗后的mPFS分别为9.67、6.67和11.87个月，与接受吉非替尼治疗的G719X/L861Q/S768I突变NSCLC患者相比获得了更好的预后。此外，有文献^[48]^报告了1例L861Q和G719X复合突变NSCLC经阿法替尼治疗后获得了32个月的PFS，预后良好。于2023年发表的ACHILLES/TORG1834是一项随机III期试验^[49,50]^，共纳入109例G719X/L861Q/S768I突变NSCLC，结果显示^[50]^，阿法替尼组的mPFS显著长于化疗组（10.6 vs 5.7个月），两组之间的ORR差别不大（61.4% vs 47.1%）。两组≥3级的不良事件（adverse event, AE）发生率分别为43.8%和37.1%。阿法替尼组中有21.9%的患者发生3级或更严重的腹泻，引起剂量减少甚至停药。值得注意的是，阿法替尼组中按照起始剂量不同分为两个亚组^[51]^，分别是30 mg/d（n=37）、40 mg/d（n=36），两组的风险比（hazard ratio, HR）分别为0.128（95%CI: 0.050-0.327）、0.704（95%CI: 0.352-1.406），差异较大。但由于各组的患者数量有限和较宽的95%CI，阿法替尼30 mg/d起始剂量是否真的劣于40 mg/d起始剂量还有待确定。Vu等^[52]^的研究表明，阿法替尼最常见的AE为腹泻（32%），皮疹次之（30%），起始剂量为40 mg/d的患者AE发生率高于起始剂量为30 mg/d的患者，但差异无统计学意义。

达克替尼（Dacomitinib）是一种强效、不可逆、高选择性的第二代EGFR-TKIs，可抑制EGFR家族所有成员的异源二聚体和同源二聚体的信号传导^[53]^。有研究^[53]^显示，32例G719X/L861Q/S768I突变NSCLC患者接受达克替尼治疗后，ORR为72.2%，mPFS为10.3个月，mOS为36.5个月。有病例报道^[54]^报告了1例达克替尼对G719X突变NSCLC患者的疗效，以起始剂量45 mg进行治疗后2个月可观察到病灶的缩小，但引起了甲沟炎，并加重至2级，因此被迫终止治疗，在甲沟炎有所改善后，以30 mg的剂量重新启动治疗，经随访仍可观察到病灶缩小。Pu等^[55]^的研究纳入了G719X（n=9）、L861Q（n=4）突变患者共13例，经达克替尼治疗后，其中G719X突变患者的ORR为66.7%（95%CI: 29.9%-92.5%），mPFS为14个月（95%CI: 4.2-23.8）；L861Q突变患者的ORR为50.0%（95%CI: 6.8%-93.2%），mPFS为6.5个月（95%CI: 0-20.7）。在治疗期间出现的AE均为1和2级，其中皮疹（87.5%）和甲沟炎（62.5%）最为常见，见表4^[6,35,42,46⇓-48,51⇓-53,55⇓⇓⇓⇓-60]^。

**表4:** 第二代TKIs在G719X/L861Q/S768I突变NSCLC中的疗效

Author	Year	Drug	Study type	n	EGFR status	ORR (%)	mPFS (mon)	mOS (mon)
Zhou et al.^[6]^	2018	Afatinib	Retrospective study	8	G719X/L861Q			49.2
Popat et al.^[35]^	2022	Afatinib	Retrospective study	94	G719X/L861Q/S768I	50.6		24.5
Ullas et al.^[42]^	2023	Afatinib	Retrospective study	4	G719X	75.0	9.7	
				2	L861Q	50.0	6.7	
				1	S768I		11.9	
Hsu et al.^[46]^	2023	Afatinib	Retrospective study	90	G719X/L861Q/S768I	63.3	17.3	28.5
Yang et al.^[47]^	2022	Afatinib	Retrospective study	181	G719X	61.3		
				97	L861Q	57.7		
				56	S768I	71.4		
Ohara et al.^[48]^	2020	Afatinib	Case report	1	L861Q+G719X		32.0	
Luo et al.^[51]^	2023	Afatinib	Ⅲ	109	G719X/L861Q/S768I	61.4	10.6	
Vu et al.^[52]^	2021	Afatinib	Retrospective study	17	G719X/L861Q/S768I	76.5		
Li et al.^[53]^	2022	Dacomitinib	Retrospective study	32	G719X/L861Q/S768I	72.2	10.3	36.5
Pu et al.^[55]^	2023	Dacomitinib	Ambispective cohort study	9	G719X	66.7	14.0	
				4	L861Q	50.0	6.5	
Li et al.^[56]^	2022	Afatinib	Retrospective study	44	G719X/L861Q/S768I	60.5	12.0	
		Dacomitinib	Retrospective study	35	G719X/L861Q/S768I	26.7	10.0	
Yang et al.^[57]^	2015	Afatinib	Ⅲ	18	G719X	78.0	13.8	26.9
				16	L861Q	56.0	8.2	17.1
				8	S768I	100.0	14.7	
Li et al.^[58]^	2021	Afatinib	Retrospective study	20	G719X	50.0		
				3	L861Q	50.0		
				12	S768I	50.0		
Park et al.^[59]^	2021	Afatinib	Retrospective study	10	G719X/L861Q		13.0	
Yang et al.^[60]^	2020	Afatinib	Retrospective study	55	G719X	63.0	14.7	
				47	L861Q	60.0	10.0	
				8	S768I	63.0	15.6	

综上，与第一代TKIs相比，EGFR G719X/L861Q/S768I突变NSCLC对第二代TKIs的敏感性较好，与经典突变相当。作为首个被美国食品药品监督管理局批准用于治疗G719X/L861Q/S768I突变NSCLC的药物，阿法替尼的药效被不断证实，但用药引起的AE或限制其更大范围的应用。Li等^[56]^的研究对阿法替尼和达克替尼在G719X/L861Q/S768I突变NSCLC中的疗效和安全性进行了比较，达克替尼组和阿法替尼组分别纳入44、35例患者。结果显示，达克替尼组的ORR显著高于阿法替尼组（60.5% vs 26.7%），达克替尼组的mPFS长于阿法替尼组（12.0 vs 10.0个月），但差异无统计学意义。达克替尼组的主要AE为皮疹（90.9%）、腹泻（77.3%）和口腔黏膜炎（61.4%），阿法替尼组的主要AE为腹泻（62.9%）、口腔黏膜炎（57.1%）和皮疹（51.4%），而且3级腹泻在阿法替尼组的发生率更高。他们还对此进行了外部队列验证，进一步说明达克替尼在G719X/L861Q/S768I突变NSCLC中的疗效优于阿法替尼，且更加安全。

### 3.4 第三代TKIs

奥希替尼（Osimertinib）是一种口服、第三代、不可逆的EGFR-TKIs，可选择性抑制EGFR敏感突变和Thr790Met（T790M）耐药突变^[61]^。有研究^[62]^表明，G719X/L861Q/S768I突变NSCLC（n=14）经奥希替尼治疗后的mPFS为10.5个月（95%CI: 3.7-15.2），mOS为13.8个月（95%CI: 7.3-29.2），收获了不错的疗效。另有一项II期临床试验^[61]^中，鉴定出的突变为G719X（n=19），其次为L861Q（n=9）、S768I（n=8），经奥希替尼治疗后，L861Q突变NSCLC的ORR为78%，其次为G719X（53%）和S768I（38%）。L861Q、G719X、S768I突变NSCLC的mPFS分别为15.2个月（95%CI: 1.3-29.1）、8.2个月（95%CI: 6.2-10.2）、12.3个月（95%CI: 0-28.8），证实G719X/L861Q/S768I突变NSCLC对奥希替尼敏感。近来的回顾性研究也验证了这一结论，Bar等^[63]^的研究结果显示，奥希替尼治疗的患者中，G719X（n=16）、L861Q（n=11）突变NSCLC的ORR分别为53%、78%，mPFS分别为8.6、15.7个月，mOS分别为18.4、25.9个月。一项回顾性研究^[64]^显示，S768I突变（n=9）对奥希替尼的敏感性最好，ORR为56%，mPFS为17个月（95%CI: 4.3-29.7）；L861Q（n=12）和G719X（n=16）相较略差，ORR分别为55%、53%，mPFS分别为11个月（95%CI: 5.8-16.2）、9个月（95%CI: 6.6-11.4）。同时，奥希替尼对G719X/L861Q/S768I复合突变也有一定疗效。1例G719X+S768I双突变患者在接受3个月的奥希替尼（80 mg/d）的治疗后实现了病灶的缩小，同时耐受性良好^[65]^。Ji等^[66]^的研究也纳入了一线使用奥希替尼的G719X/L861Q/S768I复合突变（n=15）患者，相比于单一的G719X（n=3）、L861Q（n=10）突变，ORR分别为38.4%、33.3%、40.0%，差异无统计学意义。Okuma等^[67]^的一项II期随机试验表明，相比于单一G719X突变（n=10），G719X复合突变（n=10）对奥希替尼更加敏感，ORR分别为30%、60%，mPFS分别为3.6、9.7个月。S768I突变中也观察到相同的结果，单一S768I突变（n=3）与S768I复合突变（n=7）的ORR分别为33.3%和57.1%，mPFS分别为3.7和9.8个月。治疗过程中最常见的AE是低白蛋白血症、贫血、血小板减少，其中7.5%的患者出现严重AE。以上研究均说明奥希替尼在G719X/L861Q/S768I突变NSCLC中显示出良好的治疗潜力。

伏美替尼（Furmonertinib）是一种治疗晚期肺癌的新型第三代EGFR-TKIs药物，是我国自主设计的国家I类创新药物，于2021年3月获批上市^[68]^。近来有1例伏美替尼成功治疗G719X/S768I复合突变NSCLC的病例报告，患者治疗4.5个月即观察到肺部病灶的缩小，且在随访期间未出现皮疹、腹泻或肝功能损害等与药物相关的AE，生活质量得到了显著改善^[69]^。

阿美替尼（Aumolertinib）是全球范围内第二个第三代EGFR-TKIs药物，已被证实对脑转移患者有较好的疗效。2022年报告的2例病例为阿美替尼（110 mg/d）治疗G719X/L861Q复合突变NSCLC提供了临床证据^[4]^。Shi等^[70]^通过体外细胞系研究，说明阿美替尼可显著降低G719X/L861Q/S768I突变的Ba/F3细胞的活力。而且通过创新的结构设计，阿美替尼对EGFR野生型细胞的选择性优于其他EGFR-TKIs，进而毒性反应更低。在观察性病例研究中阿美替尼也表现出较好的安全性和耐受性，即使在165 mg/d的剂量下，所有患者均未出现严重的AE和与治疗相关的剂量减少和中断。

贝福替尼（Befotertinib）是由奥希替尼的分子结构衍生而来，经国家药品监督管理局正式批准用于治疗局部晚期或转移性NSCLC的第三代EGFR-TKIs，其在分子结构设计上进行了优化，将吲哚氮原子上的甲基替换为三氟甲基，在体内更加稳定，减少了腹泻等胃肠道AE的发生^[71]^。Zhang等^[71]^于2024年报道了1例G719X/S768I复合突变患者接受贝福替尼（75 mg/d）治疗后获得部分缓解，并且无严重AE发生。目前尚缺乏更大样本的研究进一步证实贝福替尼在G719X/L861Q/S768I突变NSCLC中的疗效。

Hao等^[72]^进行了一项研究，对阿法替尼和第三代TKIs（包含奥希替尼、伏美替尼、阿美替尼）在G719X/L861Q/S768I突变NSCLC中的疗效和安全性进行了比较，结果显示阿法替尼组和第三代TKIs组的mPFS分别为13.3和11.0个月，mOS分别为30.6和24.6个月，差异无统计学意义。阿法替尼组的患者出现3级皮疹7例（11.1%），4级皮疹1例（1.6%），而第三代TKIs组中无严重AE（≥3级）的报道。这说明第三代TKIs与阿法替尼疗效相当且安全性更好，见表5^[4,61⇓⇓⇓⇓⇓-67,69,71]^。

**表5:** 第三代TKIs在G719X/L861Q/S768I突变NSCLC中的疗效

Author	Year	Drug	Study type	n	EGFR status	ORR (%)	mPFS (mon)	mOS (mon)
Li^[4]^	2022	Aumolertinib	Case report	2	G719X+L861Q		13.0	
Cho et al.^[61]^	2020	Osimertinib	II	19	G719X	53.0	8.2	
				9	L861Q	78.0	15.2	
				8	S768I	38.0	12.3	
Villaruz et al.^[62]^	2023	Osimertinib	II	14	G719X/L861Q/S768I		10.5	13.8
Bar et al.^[63]^	2023	Osimertinib	Retrospective study	16	G719X	53.0	8.6	18.4
				11	L861Q	78.0	15.7	25.9
Pizzutilo et al.^[64]^	2024	Osimertinib	Retrospective study	16	G719X	53.0	9.0	
				12	L861Q	55.0	11.0	
				9	S768I	56.0	17.0	
Morimoto et al.^[55]^	2023	Osimertinib	Case report	1	G719X+S768I			
Ji et al.^[66]^	2023	Osimertinib	Retrospective study	3	G719X	33.3		
				10	L861Q	40.0		
				14	G719X/L861Q/S768I compound mutations	38.4		
Okuma et al.^[67]^	2024	Osimertinib	II	10	G719X	30.0	3.6	
				10	G719X compound	60.0	9.7	
				3	S768I	33.3	3.7	
				7	S768I compound	57.1	9.8	
				8	L861Q	75.0	22.7	
Pan and Shi^[69]^	2024	Furmonertinib	Case report	1	G719X+S768I			
Zhang et al.^[71]^	2024	befotertinib	Case report	1	G719X+S768I			

### 3.5 联合治疗

迄今为止，研究表明免疫检查点抑制剂（immune checkpoint inhibitors, ICIs）在EGFR突变NSCLC中作用有限^[8]^。但Chen等^[73]^的研究表明，经ICIs治疗后，经典EGFR突变患者的mPFS明显短于罕见EGFR突变患者（20号外显子插入突变或G719X），分别为 1.82、2.50个月，这说明罕见EGFR突变患者可能从ICIs治疗中受益。此外，ICIs联合抗血管生成治疗在IMpower-150^[74]^和Orient-31^[75]^研究中被证实对EGFR突变的TKIs耐药NSCLC患者有良好的疗效，但这两项研究中都只包含了极少数的G719X/L861Q/S768I突变患者，且没有进一步的分析。Trummer等^[76]^对阿替利珠单抗（Atezolizumab）、贝伐珠单抗（Bevacizumab）、卡铂（Carboplatin）、紫杉醇（Paclitaxel）四药联合，即ABCP方案在EGFR突变NSCLC中的疗效进行了研究，罕见突变组纳入了3例G719X突变、1例L861Q突变及3例G719X/L861Q复合突变患者，与整个队列相比，ORR为71.4% vs 81.3%。治疗过程中有31.3%的患者发生≥3级AE，25%的患者因黏膜炎、中性粒细胞减少等原因需药物减量。目前ICIs联合血管治疗在EGFR G719X/L861Q/S768I突变NSCLC中展示出一定的应用前景，但缺乏更大样本的相关研究，其有效性和安全性尚需进一步证明。

另外，有研究^[77]^表明EGFR-TKIs联合化疗可以增强抗肿瘤活性，其活性增强的机制被解释为细胞毒性药物诱导的细胞损伤导致了EGFR表达的上调。Tamiya等^[78]^报道了1例G719X突变的患者在奥希替尼耐药后，使用阿法替尼（40 mg）和贝伐珠单抗（15 mg/kg）联合治疗，耐受良好且症状持续好转。同样，EGFR-TKIs联合化疗应用于G719X/L861Q/S768I突变NSCLC的研究较少，其有效性尚不能被明确证明。

### 3.6 小结

根据Heymach团队于2021年发表的一项基于EGFR基因结构的研究^[9]^，L861Q突变的位点远离ATP结合口袋，因此对药物结合几乎没有影响，对EGFR-TKIs具有泛敏感性。而G719X和S768I均属于PACC突变，P环方向的改变使奥希替尼的吲哚环无法接近P环，导致了药物结合的不稳定，从而疗效不佳。而第二代EGFR-TKIs作用的机制是在铰链区与主链形成氢键，并与疏水区相互作用，位点与P环无关，故药物结合不受影响，因此从结构上来说，第二代EGFR-TKIs对此类突变的疗效更好^[9,79]^。

在真实世界的病例研究中，G719X/L861Q/S768I突变 NSCLC对第一代TKIs的敏感性差于经典突变。第二代TKIs和第三代TKIs在G719X/L861Q/S768I突变NSCLC中的疗效相当，且第三代TKIs的安全性更好。新型第三代TKIs，如阿美替尼、伏美替尼、贝福替尼的研发，可能为G719X/L861Q/S768I突变 NSCLC的治疗提供更多选择。此外，ICI联合化疗及EGFR-TKI联合化疗在G719X/L861Q/S768I突变 NSCLC的治疗中也展现出不错的前景，但其有效性和安全性尚需更大样本的研究证实。

## 4 总结与展望

随着基因检测技术的进展，罕见EGFR突变的检出率大幅提高，其中最主要的为G719X、L861Q、S768I，分别占~3%、~1%、~1%^[6]^。与此同时，不同罕见突变对TKIs治疗敏感性的异质性也得到了关注，对罕见突变的治疗进展有一定的推动作用。考虑到治疗疗效的监测及肿瘤晚期患者需要重复检测基因突变，创伤较小的ctDNA检测已得到广泛应用，检测的敏感度、准确性也得到证实。相比于第一代TKIs，G719X/L861Q/S768I突变NSCLC对第二代和第三代TKIs具有更高的敏感性。新型的第三代TKIs及联合治疗在G719X/L861Q/S768I突变NSCLC中的应用均有相关病例报道，显示出不错的治疗前景。但由于G719X/L861Q/S768I的突变率较低，纳入研究的样本数较少，且大多数研究均为回顾性研究，故需要更大样本的前瞻性研究说明不同TKIs的疗效及安全性，同时，有关联合治疗的应用也需要进一步的探索。期待在未来的研究中有更多G719X/L861Q/S768I突变NSCLC诊断及治疗的进展。
